# Complete mitochondrial genome of *Biston thoracicaria* (Lepidoptera: Geometridae)

**DOI:** 10.1080/23802359.2021.1939181

**Published:** 2021-06-17

**Authors:** Biao-Sheng Huang, You-Jie Zhao, Yu-Peng Wu

**Affiliations:** a College of Big data and Intelligent Engineering, Southwest Forestry University, Kunming, China; b College of Environment and Safety, Taiyuan University of Science and Technology, Taiyuan, China

**Keywords:** Geometridae, Biston thoracicaria, mitogenome, phylogeny

## Abstract

The complete mitochondrial genome (mitogenome) of *Biston thoracicaria* (Lepidoptera: Geometridae) is 15,538 bp in length, containing 13 PCGs, 22 tRNAs, two rRNAs, and an A + T-rich region. All PCGs initiate with typical start codon of ATN and share the complete stop codon of TAA, whereas *cox1* starts with CGA. The ML analysis was performed using a dataset matrix containing 13 PCGs concatenated from the mitogenomes of Geometridae species. Our study presented the phylogenetic relationship of (Larentiinae + ((Sterrhinae + (Ennominae + Geometrinae))). Within the genera *Biston*, *B. thoracicaria* grouped with other species as the sister group.

Geometridae is the second largest family of Lepidoptera, including about 35,000 species throughout the world (Scoble 1999). They are considered as the economically important pests of woody plants. Some species also play important roles in genetic and evolutionary biology. For example, the peppered moth (i.e., *Biston betularia*) was a typical example for evolutionary study in 20th century, allowing biologists to solve questions about gene flow, rates of selection, and the neutral theory (Cook and Saccheri 2013). In this study, we presented the complete mitogenome of *Biston thoracicaria* Oberthür, 1884, which would facilitate the further understanding of the phylogenetic relationship and phylogeography of both *Biston* and Geometridae.

The specimen was collected from Taiyuan City of China (N37.833393, E112.666114) in July 2020, and the species was identified by Wu Yupeng from Taiyuan University of Science and Technology. A specimen was deposited at Herbarium of Institute of Plant Protection, Shanxi Academy of Agricultural Sciences (http://www.sxagri.ac.cn, Wu Yupeng and wuyupeng007@163.com) under the voucher number 20200540. Total genomic DNA was extracted from a single female with the QIAamp DNA Mini Kit (QIAGEN, Hilden, Germany) following the manufacturer’s instruction. The sample was sequenced by Illumina HiSeq 4000 in Novogene (Tianjin, China), with a read length of 150 bp and the size of raw data 7.1 Gb (GenBank accession No. SRR13958261). The mitogenome was assembled using GetOrganelle v1.6.4 program (the options: -F animal_mt -R 15 -k 145) (Jin et al. 2020) and annotated by MITOS2 webserver (Bernt et al. 2013).

The complete mitogenome of *B. thoracicaria* (GenBank accession No. MN956510) is a circular molecule of 15,538 bp in length. It contains the typical set of 37 genes as in most insect mitogenomes (Cameron 2014), including 13 protein-coding genes (PCGs), 22 tRNAs, 2 rRNAs genes, and an A + T-rich region. All genes present the identical order and orientation with those of most other mitogenomes of Lepidoptera (Wang et al. 2016, Wu et al. 2016). The J-strand encodes most of the genes (14 tRNAs and nine PCGs), while the other genes (eight tRNAs, two rRNAs, and four PCGs) are located on the N-strand. The overall base composition is 40.3% A, 41.2% T, 11.1% C, and 7.4% G. All PCGs use the standard start codon of ATN and the common stop codon of TAA, except that *cox1* starts with CGA. The *rrnL* is located between *trnL1* and *trnV*, with the length of 1399 bp. The *rrnS* is located between *trnV* and the A + T-rich region, with the length of 778 bp. The control region is 446 bp in length, and is located between *rrnS* and *trnM*.

Phylogenetic analysis was performed on concatenated nucleotide sequences of 13 PCGs derived from 18 Geometridae species, with three Larentiinae species as the outgroups. Phylogenetic inference was conducted using IQ-TREE 1.6.12 (Nguyen et al. 2015), with 1000 replicates of ultrafast likelihood bootstrap. The phylogenetic relationships among subfamilies were as follows: (Larentiinae + ((Sterrhinae + (Ennominae + Geometrinae))) (Figure 1), which were generally consistent with most other studies (Abraham et al. 2001; Ding et al. 2020). Within the genera *Biston*, *B. thoracicaria* grouped with other species as the sister group.

**Figure 1.**
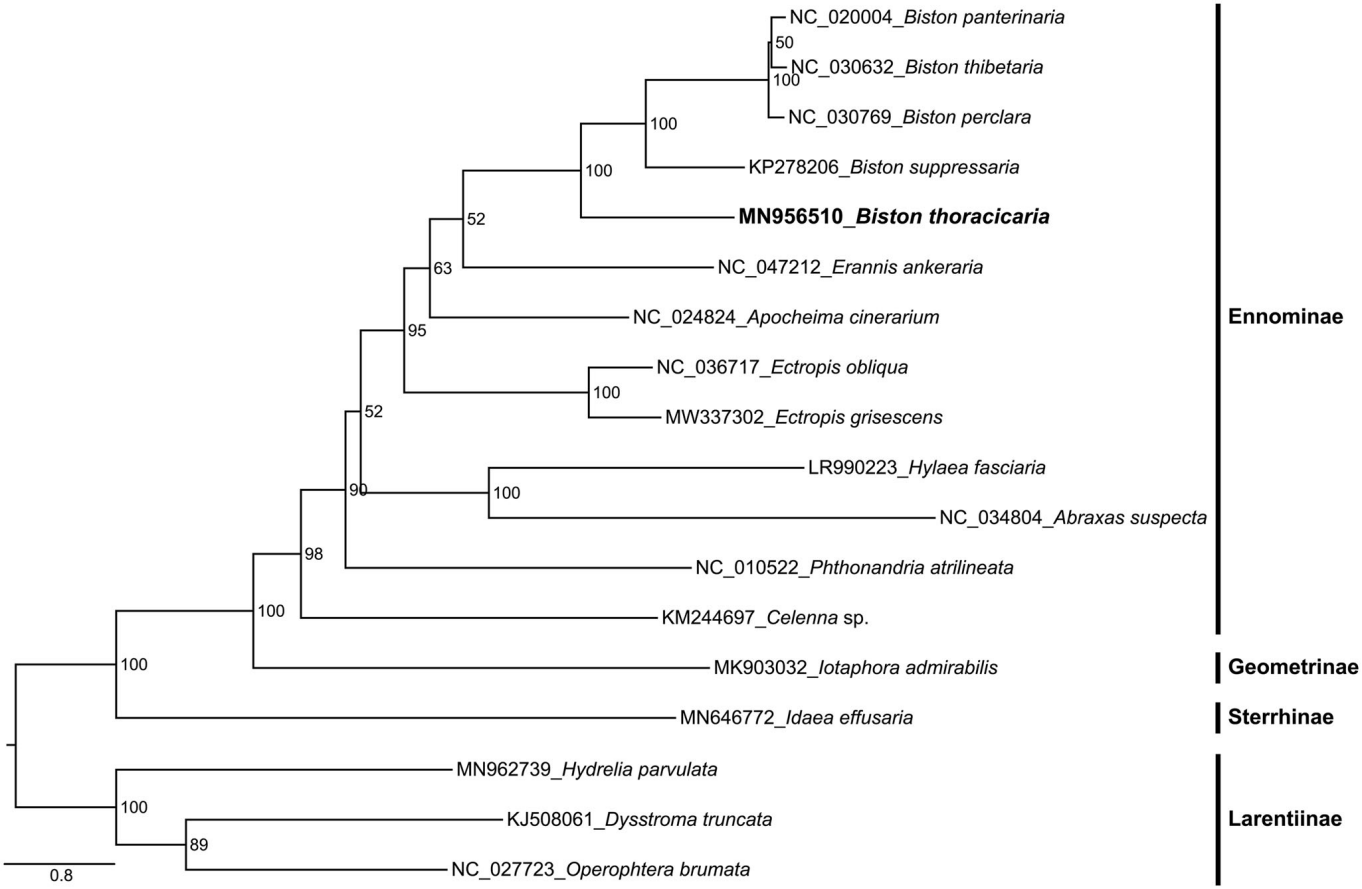
The maximum-likelihood (ML) phylogenetic tree of 18 Geometridae species inferred from the concatenated 13 PCG nucleotide sequence data.

## Data Availability

The data of this study are openly available in NCBI (National Center for Biotechnology Information) at https://www.ncbi.nlm.nih.gov/, reference number MN956510.
